# Supplementary material for: Risk factors of HIV infection among female entertainment workers in Cambodia: Findings of a national survey
Source: PLoS One. 2020 Dec 21;15(12):e0244357. doi: 10.1371/journal.pone.0244357 (PMC7751854; doi:10.1371/journal.pone.0244357)
Supplement: S1 Questionnaire — (PDF) [file pone.0244357.s001.pdf]

PIN:

Stick pre-printed label of study participant

Integrated Bio-Behavioral Survey (IBBS) 2016:
Female Entertainment Workers (FEW)

Informed Consent and Voluntary Participation

I have read and explained the Informed Consent document to the participant. She understands the process of the study, objectives, potential risks, and benefits and that her participation in the study is voluntary. The participant explained the study activities back to me, and I believe that she understood what is going to happen in this study. She gives her informed consent verbally.

Province number:

1. PHNOM PENH
2. KAMPONG CHAM – THBONG KHMUM
3. PREY VENG
4. BATTAMBANG
5. BANTEY MEANCHEY
6. SIEM REAP
7. KOH KONG
8. SIHANOUK VILLE
9. PURSAT
10. KANDAL
11. KAMPONG THOM
12. KAMPONG CHHNANG
13. TAKEO
14. KAMPONG SPEU
15. OTDAR MEANCHEY
16. PREAH VIHEAR
17. PAILIN
18. RATTANAKIRI

Cluster code:

CODE

FEW type:

1. Former brothel
2. Freelance
3. Beer promoter
4. Karaoke/Massage
5. Beer Garden

Did the interviewee abandon the interview? ☐ YES, (at question #) **Q**

☐ NO

Interviewer's name:

Date interview: / / 2015

Interviewer's signature: ____________________

Checked by the team leader: Signature…………………..… Date: / / 2015

SECTION 1: SOCIO-DEMOGRAPHIC CHARACTERISTICS

N⁰	QUESTIONS	CODING CATEGORIES	Skip to	CODE
**1**	How old are you?<br>(in completed years)	Number of years……….<br>(18-49 y.o)		
**2**	How many years of school have you completed now?	Number of years……….<br>(No school: 0)		
**3**	Are you married?<br><br>***Read out all the answers. Participant can choose only one response.***	Never married:0<br>Yes, married and living together: 1<br>Yes, married but not living together: 2<br>Divorced/ Widowed: 3<br>Not married, but living with partner: 4		
**4**	For how long have you been living in (<u>name of the city/town where EW works</u>)?<br>(in completed months)	Number of months……….<br>Less than 1 month:1<br>More than 12 months: 13		
**5**	In how many different cities/ towns, including this one, have you been living in the past 12 months?	Number of cities……..<br>Not move: 0		
**6**	For how many months have you been working at your current place of work?<br>(in completed months)	Number of months……...<br>Less than a month: 0<br>More than 12 months: 13		
**7**	Before coming to work on this job, did you used to work any of the following jobs?<br><br>***Read out all the answers. The participant can choose several.***	Dancer at night club: 1<br>Massager: 2<br>Beer promotion girl: 3<br>Worked at karaoke parlor: 4<br>Worked at beer garden (but not beer promotion): 5<br>Worked at a restaurant: 6<br>Sex worker at brothel: 7<br>Freelance : 8<br>Garment worker: 9<br>None of the above: 99		
**8**	How old were you when you first had sexual intercourse?	Number of years……..<br>Never had sexual intercourse: 0		
**9**	Since you started working as an entertainment worker (***any type of EW, not just current job***), how many times did you get pregnant?	Number of pregnancies…….<br>Never got pregnant: 0	→ **14**	
**10**	How many children do you have now?	Number of children…….<br>No child: 0		
**11**	While working as an entertainment worker (***any type of EW, not just current job***), how many abortions have you had?	Number of abortion……..<br>Never had abortion: 0	→ **14**	

**12**	How many months ago was your last abortion?<br><br>***Clarify: Not how many months pregnant at the time of the abortion.***	Number of months……..		
**13**	Where did you go for last abortion?<br><br>***Participant can choose only one response.***	Private clinics: 1<br>Traditional practitioner: 2<br>Health center/ public hospital: 3<br>NGO clinic: 4<br>Bought drugs from pharmacy: 5<br>Other, *specify:*……………………………: 6		

SECTION 2: RISK BEHAVIORS

N^0	QUESTIONS	CODING CATEGORIES	Skip to	CODE
**14**	In the past 12 months, how many boyfriends / sweethearts did you have?	Number……..<br>None: 0	 → **19**	
**15**	In the past 12 months, did you have sex with your boyfriend / sweetheart?	Yes: 1<br>No: 2	 → **19**	
**16**	In the last month, how many times did you have sex with a boyfriend/ sweetheart (s)?	Number……<br>None: 0		
**17**	In the past three month, how many times did you have sex with a boyfriend/ sweetheart (s)?	Number……<br>None: 0		
**18**	For the last 3 months, how often did you use condoms with your boy-friend/ sweetheart (last or current sweetheart)?	Never had sex: 0<br>All the time (100%): 1<br>Frequently (60-99%): 2<br>Sometimes(1-59%): 3<br>Never (0%): 0		
**19**	For the past 3 months, how often have you used condoms with your husband?	Never had sex: 0<br>All the time (100%): 1<br>Frequently (60-99%): 2<br>Sometimes(1-59%): 3<br>Never (0%): 0		
**20**	For the past 12 months, have you ever received gifts or money from men in exchange for sex? (<u>not</u> including boyfriend/ sweetheart)	Never had sex: 0<br>Yes: 1<br>No: 2	→ **35**<br><br>→ **30**	
**21**	How many clients, on average, do you have sex with in a regular week? (<u>not</u> including your boyfriend or sweetheart)	Number of clients<br>None: 0		
**22**	How many clients did you have sex within your last working day?	Number of clients……<br>None: 0		

	**(During Last 12 months)**			
**23**	Did you use a condom with your most recent client? **(During Last 12 months)**	Yes: 1<br>No: 2		
**24**	For the last week, how often did you use condoms with your clients?	Did not have sex with client: 0<br>All the time (100%): 1<br>Frequently (60-99%): 2<br>Sometimes(1-59%): 3<br>Never (0%): 4		
**25**	For the last 3 months, how often did you use condoms with your clients?	Did not have sex with client: 0<br>All the time: 1<br>Frequently: 2<br>Sometimes: 3<br>Never: 4		
**26**	Currently, do you have regular sex clients (sleeping with you more than five times)?	Yes: 1<br>No: 2	 → **28**	
**27**	For the last one month, how regularly have you used condoms with regular clients?	Never had sex with client: 0<br>All the time (100%): 1<br>Frequently (60-99%): 2<br>Sometimes(1-59%): 3<br>Never (0%): 4		
**28**	In the past 12 months, did you have sex with a financial supporter?	No financial supporter: 0<br>Yes: 1<br>No: 2	→ **30**<br><br>→ **30**	
**29**	For the last 3 months, how often did you use condoms with your financial supporter (last or current)?	Never had sex: 0<br>All the time (100%): 1<br>Frequently (60-99%): 2<br>Sometimes(1-59%): 3<br>Never (0%): 4		
**30**	In the past 3 months did you have sex in the anus?	Never had sex: 0<br>Yes: 1<br>No: 2		
**31**	In the past 3 months did you give oral sex?	Never had sex: 0<br>Yes: 1<br>No: 2		
**32**	In the past 3 months, did you ever have sex without using a condom beside your husband?	Never had sex/never had sex with client : 0<br>Yes: 1<br>No: 2	→ **35**<br><br>→ **35**	
**33**	If you didn't use condoms during sex, why did you have unprotected sex?<br><br>***Read out all the answers. The participant can choose several.***	I wanted to become pregnant: 1<br>I thought he didn't have a STI or HIV: 2<br>He persuaded me not to use condoms: 3<br>He forced me not to use condoms: 4<br>He offered more money for sex without: 5<br>Faithful/Understand each other: 6<br>Condom not available: 7<br>I was impaired by drugs or alcohol: 8<br>Other ______________________________: 9		
**34**	This past month, how many days did you stop having sex during your menstruation?	Number of days……<br>Did not stop : 0<br>Never had sex: 96		

		Didn't have menstruation: 97		
**35**	In the past 3 months, did you receive any education about HIV or STI?<br><br>Read out all the answers. The participant can choose several.	Did not get any educational info: 0<br>TV: 1<br>Radio: 2<br>Newspaper: 3<br>Billboard: 4<br>Poster: 5<br>Booklet/ publication: 6<br>Group discussion with outreach worker: 7<br>Individual Counseling: 8<br>Peer educator: 9<br>Other ___________________________:10		
**36**	For the last 3 months, from whom did you receive educational information about HIV/AIDs and STIs?<br>***Read out all the answers. The participant can choose several.***	Never got any educational info: 1<br>STI clinic staff: 2<br>NGO clinic staff: 3<br>Outreach worker: 4<br>Peer Educator (friends educating friends): 5<br>Staff of VCCT: 6<br>Private clinic physicians or staff: 7		
**37**	In the last 12 months, have you been given condoms?	Yes: 1<br>No: 2		
**38**	In the past 3 months, did you buy condoms or receive condoms for free?	Did not use condoms: 0<br>Always got condoms for free: 1<br>Sometimes free, sometimes bought: 2<br>Always bought condoms: 3<br>Got condoms from Client/boyfriend: 4<br>Never receive free condom: 5		
**39**	In the past 3 months, from where did you get condoms?<br><br>***Read out all the answers. The participant can choose several.***	Did not use condoms: 0<br>At the place I work: 1<br>From the client: 2<br>From an outreach worker: 3<br>From a peer educator: 4<br>From a pharmacy or store: 5<br>Family health clinic/ HC/ public hospital: 6<br>NGO clinic: 7<br>Other ___________________________: 8		

Section 3: RISK FACTORS FOR HIV – PHYSICAL AND SEXUAL VIOLENCE

N0	QUESTIONS	CODING CATEGORIES	Skip to	CODE
**40.a**	Did your husband/ partner ever hit, slap, kick or do anything else to hurt you physically?	Yes: 1<br>No: 2<br>No response: 98		
**40.b**	How often did this happen during the last 12 months: often, only sometimes, or not at all?	Often: 1<br>Sometimes: 2<br>Not at all: 3<br>No response: 98		

**41.a**	Did someone other than your husband/partner ever hit, slap, kick or do anything else to hurt you physically?	Yes: 1<br>No: 2<br>No response: 98		
**41.b**	How often did this happen during the last 12 months: often, only sometimes, or not at all?	Often: 1<br>Sometimes: 2<br>Not at all: 3<br>No response: 98		
**42.a**	Have you ever been physically forced by your husband/partner into having sexual intercourse with him when you did not want to?	Yes: 1<br>No: 2<br>No response: 98		
**42.b**	How often did this happen during the last 12 months: often, only sometimes, or not at all?	Often: 1<br>Sometimes: 2<br>Not at all: 3<br>No response: 98		
**43.a**	Have you ever been physically forced by someone other than your husband/partner into having sexual intercourse with him when you did not want to?	Yes: 1<br>No: 2<br>No response: 98		
**43.b**	How often did this happen during the last 12 months: often, only sometimes, or not at all?	Often: 1<br>Sometimes: 2<br>Not at all: 3<br>No response: 98		
**44.a**	Have you ever been physically forced by a group of men* into having sex with them when you did not want to?<br>*Two or more men	Yes: 1<br>No: 2<br>No response: 98		
**44.b**	How often did this happen during the last 12 months: often, only sometimes, or not at all?	Often: 1<br>Sometimes: 2<br>Not at all: 3<br>No response: 98		
	If “yes” to any of questions 40-44, please continue:			
**45**	Did you seek medical help or treatment for injuries cause by forced sexual intercourse or any other forced sexual acts?	Yes: 1<br>No: 2<br>No response: 98		

Section 4: DRUG USE

N⁰	QUESTIONS	CODING CATEGORIES	Skip to	CODE
46	Some people have used drugs for various reasons such as for fun and to boost their energy, have you ever used drugs? ***Remind the client that her answers are private and confidential.***	Yes: 1<br>No: 2<br>No response: 98<br>Don't know: 99	 → 49	
47	For the past 12 months, which type of drugs have you used? (Please read out the answers, there can be many)	Never used drugs past year: 0<br>Heroin: 1<br>Amphetamine (Ice, Yama): 2<br>Ecstasy: 3<br>Sleep booster (Valium,Diazepam): 4<br>Glue: 5<br>Marijuana: 6<br>Other drugs ________________________: 7<br>No response: 98		
48	For the past 12 months, have you injected drugs?	Yes: 1<br>No: 2<br>No response: 98<br>Don't know: 99		
49	In the past week, how often did you drink alcohol while working?	Number of days……<br>None: 0<br>Every day: 7		
50	In the past week, how many drinks do you usually consume per week while working?<br>*(A drink is: a can or bottle of beer, a glass of wine, a shot of alcohol)*	Number of drinks..........<br>None: 0<br>96 drinks or more: 96<br>Less than one drink: 97		

SECTION 5: HEALTH SEEKING BEHAVIORS

N⁰	QUESTIONS	CODING CATEGORIES	Skip to	CODE
51	In the past 12 months, have you ever had genital ulcer/sore(s)?	Yes: 1<br>No: 2		
52	In the past 12 months, have you ever had genital warts?	Yes: 1<br>No: 2		
53	In the past 12 months, have you ever had an abnormal vaginal discharge?	Yes: 1<br>No: 2		
54	In the past 12 months, have you ever had lower abdominal pain?<br>***Point to the lower abdomen/ pelvic area***	Yes: 1<br>No: 2		
55	In the past 12 months, do you think that you had a	Yes: 1<br>No: 2		

	sexual disease?			
**56**	In the past 12 months, where did you get treatment last time you had a sexual disease?	Never had sexual disease: 0<br>Public hospital / health center: 1<br>Family health clinic (public STD clinic): 2<br>NGO run STD clinic: 3<br>Private hospital / doctor: 4<br>Traditional practitioner: 5<br>Bought drugs from pharmacy: 6<br>No treatment at all: 7<br>Other ______________________________: 8	→ **58**	
**57**	For how long did you stop having sex during your last episode of sexual disease?<br><br>***Read out all the answers.*** **Participant can choose only one response.**	Never had sex: 0<br>Did not stop having sex: 1<br>One to 3 days: 2<br>4 days to 10 days: 3<br>Until completed treatment: 4<br>Until cured: 5		
**58**	Currently, what means or methods do you use to prevent pregnancy?<br><br>***Read out all the answers.*** **Participant can choose several answers.**	Not having sex/ Never had sex: 0<br>None/ do not use any methods: 1<br>Intrauterine contraceptive device: 2<br>Male sterilization: 3<br>Daily oral contraceptive pills: 4<br>Monthly oral contraceptive pills: 5<br>Injectables: 6<br>Intrauterine device (IDU) : 7<br>Female sterilization:8<br>Male Condoms: 9<br>Female Condoms: 10<br>Lactational amenorrhea:11<br>Spermicide: 12<br>Period-based/ Rhythm: 13<br>Withdrawal: 14<br>Emergency contraception / postcoital pill: 15<br>Other…………….: 16		

SECTION 6: KNOWLEDGE OF HIV and STI STATUS

N^0	QUESTIONS	CODING CATEGORIES	Skip to	CODE
**59**	Do you know where you can go if you wish to receive an HIV test?	Yes: 1<br>No: 2		
**60**	For the past 12 months, have you ever had a blood test for HIV?	Yes: 1<br>No: 2		
**61**	If you ever had a blood test for HIV, how many months ago did you have the test?	Months……<br>Never had a blood test: 0<br>Less than 1 month or 1 month: 1<br>More than a year ago: 13		
**62**	Where did you have the very last blood test for HIV?	Never had a blood test: 0<br>Private clinic or laboratory: 1<br>Public hospital: 2<br>VCCT (government): 3	→ **69**	

		VCCT (NGO): 4<br>Mobile HIV testing: 5<br>Pharmacy: 6<br>Others__________________________: 7		
**63**	Did you receive the result of your last blood test for HIV? You don't have to tell me the result.	Yes: 1<br>No: 2		
**64**	Do you feel comfortable telling me the result of your last HIV test?	Yes: 1<br>No: 2	 → **69**	
**65**	If you feel comfortable telling me, what was your test result?<br>**Remind participant that her answers are confidential.**	HIV positive: 1<br>Intermediate: 2<br>HIV negative: 3	 <br>→ 69	
**66**	If your test is positive, how many times did you test to confirm your status?	Number of times….		
**67**	If your test is positive, had you registered at any OI/ARV site?	Yes: 1<br>No: 2		
**68**	If your test is positive, are you receiving ARV drugs?	Yes: 1<br>No: 2		
**69**	Are ARV services available in Cambodia for HIV/AIDS-infected people?	Yes, ARV services are available: 1<br>No, ARV services are not available: 2<br>I don't know if ARV services are available: 3		
**70**	For the past 12 months, how often have you visited a clinic for sexual health check-ups?	Number of visits…….<br>None in the past year: 0	 → **73**	
**71**	How many months ago was your last visit to a clinic for a sexual health check-up?	Months…..<br>None in the past year: 0		
**72**	What was the clinic you visited?<br><br>***Read out all the answers.* Participant can choose several answers.**	Family Health Clinic (run by NCHADS): 1<br>Other government/ MoH clinic: 2<br>NGO clinic run by RHAC, MSIC, MEC, PSF: 3<br>NGO clinic run by other organization: 4<br>Private clinic: 5<br>Don't know which organization ran clinic: 6<br>Other type of clinic__________________: 7		
**73**	When you have sexual intercourse with only one HIV/AIDS-negative faithful partner, can this transmit HIV/AIDS?	Don't know: 0<br>Yes: 1<br>No: 2		
**74**	Can condoms prevent HIV/AIDS transmission?	Don't know: 0<br>Yes: 1<br>No: 2		

**75**	Can a healthy-looking person transmit HIV/AIDS infection?	Don't know: 0<br>Yes: 1<br>No: 2		
**76**	Can mosquitoes transmit HIV/AIDS from people infected with HIV/AIDS to other people without HIV/AIDS?	Don't know: 0<br>Yes: 1<br>No: 2		
**77**	Can eating with HIV/AIDS-infected people transmit HIV/AIDS infection?	Don't know: 0<br>Yes: 1<br>No: 2		

END

Read aloud: Thank you for participating in the survey and answering these questions.
