# Supplementary material for: Risk factors of HIV infection among female entertainment workers in Cambodia: Findings of a national survey
Source: PLoS One. 2020 Dec 21;15(12):e0244357. doi: 10.1371/journal.pone.0244357 (PMC7751854; doi:10.1371/journal.pone.0244357)
Supplement: S2 Questionnaire — (PDF) [file pone.0244357.s002.pdf]

លេខកូដ

បិតលេខកូដរបស់អ្នកចូលរួម ដែលព្រីនទុកជាមុន

តារាងសំណួរសម្រាប់ការអង្កេតតាមដាន ចំណេះដឹង ស្តីពីជំងឺអេដស៍ ការប្រព្រឹត្តផ្លូវភេទ និងកត្តាប្រឈមនឹងការឆ្លងមេរោគអេដស៍ ឆ្នាំ២០១៦ ក្នុងចំណោមស្ត្រីបំរើសេវាកំសាន្តសប្បាយ នៅក្នុងប្រទេសកម្ពុជា

ការចូលរួមដោយស្ម័គ្រចិត្តនិងសុំការយល់ព្រម

ខ្ញុំបានអាននិងពន្យល់ទម្រង់សុំការយល់ព្រមទៅអ្នកចូលរួម។ គាត់បានយល់ពីដំណើរការនៃការសិក្សា គោលបំណង ហានិភ័យដែលអាចកើតមាន និងអត្ថប្រយោជដែលទទួលបាន និងបញ្ជាក់ថាគាត់ចូលរួមការសិក្សានេះដោយស្ម័គ្រចិត្ត។ អ្នកចូលរួមបានពន្យល់សកម្មភាពសិក្សាត្រឡប់មកខ្ញុំវិញ ខ្ញុំជឿជាក់ថាគាត់បានយល់ពីអ្វីដែលនឹងកើតឡើងនៅក្នុងការសិក្សានេះ។ គាត់ផ្តល់ការយល់ព្រមចូលរួមដោយផ្ទាល់មាត់។

លេខខេត្ត:

១- ភ្នំពេញ	៧- កោះកុង	១៣- តាកែវ
២- កំពង់ចាម-ត្បូងឃ្មុំ	៨- ព្រះសីហនុ	១៤- កំពង់ស្ពឺ
៣- ព្រៃវែង	៩- ពោធិសាត់	១៥- ឧត្តរមានជ័យ
៤- បាត់ដំបង	១០- កណ្តាល	១៦- ព្រះវិហារ
៥- បន្ទាយមានជ័យ	១១- កំពង់ធំ	១៧- ប៉ៃលិន
៦- សៀមរាប	១២- កំពង់ឆ្នាំង	១៨- រតនៈគីរី

លេខចង្កោម (ក្លាស្ទ័រ):

ប្រភេទស្ត្រីបម្រើសេវាកំសាន្តសប្បាយ :

១. នារីបំរើការងារនៅតាមអតីតផ្ទះបន លេខកូដ

២. នារីបំរើសេវាកំសាន្តឯករាជ្យ

៣. នារីបំរើស្រាបៀរ

៤. នារីបំរើការ៉ាអូខេ/ម៉ាស្សា

៥. នារីបំរើសេវានៅបៀរហ្គាឌិន/ភោជនីយដ្ឋាន

តើអ្នកចូលរួមបោះបង់ចោលការសំភាសន៍ដែរឬទេ?

១. បោះបង់ចោល (នៅសំនួរទី) សំនួរ

២. មិនបានបោះបង់ចោល

ឈ្មោះអ្នកសំភាសន៍: ………………………………………..

ហត្ថលេខាអ្នកសម្ភាស ______________________

កាលបរិច្ឆេទ: .... / ……………….. /២០១៥

បានពិនិត្យផ្ទៀងផ្ទាត់ដោយប្រធានក្រុមការងារ: ហត្ថលេខា.........................ថ្ងៃទី......ខែ......ឆ្នាំ២០១៦

ផ្នែកទី១: លក្ខណប្រជាសាស្ត្រសង្គម

លរ	សំណួរ	កូដ ចម្លើយ	រំលង	កូដ
១	តើអ្នកមានអាយុប៉ុន្មាន? (អាយុពេញឆ្នាំ)	អាយុ.................. ឆ្នាំ<br>(អាយុ ១៨ ទៅ ៤៩ ឆ្នាំ)		
២	តើនាងរៀននៅសាលាបានប៉ុន្មានឆ្នាំ?	ចំនួន.......................ឆ្នាំ<br>មិនបានរៀន: ០		
៣	តើនាងរៀបការហើយឬនៅ? *អានចម្លើយទាំងអស់។ អ្នកចូលរួមអាចជ្រើសរើសបានតែមួយទេ*	មិនដែលរៀបការ: ០<br>រៀបការហើយនិងកំពុងរស់នៅជាមួយគ្នា: ១<br>រៀបការហើយតែរស់នៅឆ្ងាយពីគ្នា: ២<br>លែងលះគ្នា/មេម៉ាយ: ៣<br>មិនបានរៀបការប៉ុន្តែរស់នៅជាមួយដៃគូ: ៤		
៤	តើនាងមករស់នៅ (ឈ្មោះ ក្រុងប្រជុំជនដែលស្ត្រីរស់នៅនិងធ្វើការងារ) អស់រយៈពេលប៉ុន្មានខែហើយ? (ខែពេញលេញ)	ចំនួន .... ខែ<br>តិចជាងមួយខែ: ១<br>លើសពី១២ខែ: ១៣		
៥	ក្នុងរយៈពេល១២ខែកន្លងមក តើមានចំនួនខេត្ត/រាជធានីប៉ុន្មាន ដែលនាងធ្លាប់បានរស់នៅ រួមទាំងខេត្ត-រាជធានីនេះផង?	ចំនួនខេត្ត-រាជធានី .... អត់បានទៅណា: ០		
៦	តើនាងធ្វើការនៅទីកន្លែងបច្ចុប្បន្ននេះ បានរយៈពេលប៉ុន្មានខែហើយ?	ចំនួន...................ខែ<br>តិចជាងមួយខែ: 0<br>លើសពី១២ខែ: ១៣		

៧	មុនពេលមកធ្វើការងារនេះ តើនាងធ្លាប់បានធ្វើការណាមួយដូចខាងក្រោមដែរឬទេ**?** មិនយកការងារដែលកំពុងធ្វើទេ (សូមអានចំលើយ, អាចមានចំលើយច្រើន)	អ្នករាំតាមរង្គសាល/ក្លិប: ១<br>អ្នកម៉ាស្សា: ២<br>អ្នកបំរើស្រាបៀរ: ៣<br>អ្នកបំរើការងារការ៉ាអូខេ: ៤<br>អ្នកបំរើការងារនៅបៀរហ្គារដិន: ៥<br>អ្នកបំរើការតាមភោជនីយដ្ឋាន: ៦<br>នារីរកស៊ីផ្លូវភេទនៅក្នុងផ្ទះបន: ៧<br>នារីបំរើសេវាកំសាន្តឯករាជ្យ: ៨<br>កម្មការិនីរោងចក្រ: ៩<br>មិនមែនការងារទាំងអស់ខាងលើ: ៩៩		
៨	តើនាងចាប់ផ្តើមរួមភេទលើកដំបូងនៅអាយុប៉ុន្មានឆ្នាំ?	អាយុ............. ឆ្នាំ<br>មិនដែលរួមភេទ: 0	ទៅសំនួរ	១៤
៩	ចាប់តាំងពីនាងធ្វើការជានារីបំរើសេវាកំសាន្ត តើនាងធ្លាប់មានផ្ទៃពោះប៉ុន្មានដង?	ចំនួនផ្ទៃពោះ................ដង<br>មិនធ្លាប់មានផ្ទៃពោះ: 0	**<u>ទៅសំនួរ</u>**	១៤
១០	បច្ចុប្បន្ននេះ តើនាងមានកូនប៉ុន្មាននាក់?	ចំនួន................កូន<br>មិនដែលបានបង្កើតកូន: 0		
១១	ក្នុងខណៈពេលដែលនាង ធ្វើការជានារីបំរើសេវាកំសាន្ត តើនាងធ្លាប់បានរំលូតកូនប៉ុន្មានដងដែរ?	ចំនួនរំលូតកូន ....<br>មិនធ្លាប់រំលូតកូន: 0	**<u>ទៅសំនួរ</u>**	១៤
១២	តើនាងបានរំលូតកូនលើកចុងក្រោយប៉ុន្មានខែកន្លងមកហើយ នៅពេលធ្វើការជានារីបំរើសេវាកំសាន្ត?	ចំនួន....................ខែ<br>តិចជាងមួយខែ: ១		
១៣	តើនាងបានទៅធ្វើការរំលូតកូនលើកចុងក្រោយនៅទីណា? (ចម្លើយមានតែមួយ)	គ្លីនិកឯកជន: ១<br>ធ្មបបូរាណ (គ្រូបូរាណ) ២<br>មណ្ឌលសុខភាព/មន្ទីរពេទ្យរដ្ឋ: ៣<br>គ្លីនិកអង្គការ: ៤		

		ទិញថ្នាំពីឱសថស្ថានមកលេប: ៥<br>ផ្សេងៗ(សូមបញ្ជាក់)……………………: ៦		

ផ្នែកទី២: ឥរិយាបទប្រឈមនឹងគ្រោះថ្នាក់

លរ	សំណួរ	កូដ ចម្លើយ	រំលង	កូដ
១៤	ក្នុងរយៈពេល១២ខែកន្លងមក តើនាងធ្លាប់មានមិត្តប្រុស/សង្សារ ប៉ុន្មាននាក់?	ចំនួន……….នាក់<br>មិនធ្លាប់មានមិត្តប្រុស/សង្សារ: ០	ទៅសំនួរ	១៩
១៥	ក្នុងរយៈពេល ១២ខែកន្លងមក តើនាងធ្លាប់បានរួមភេទ ជាមួយ មិត្តប្រុស/សង្សារ ដែរ ឬទេ?	ធ្លាប់: ១<br>មិនធ្លាប់: ២	ទៅសំនួរ	១៩
១៦	រយៈពេល ១ខែកន្លងមកនេះ តើនាងធ្លាប់បានរួមភេទ ជាមួយមិត្តប្រុស/សង្សាររបស់នាងប៉ុន្មានដងដែរ?	ចំនួន ……………. ដង<br>មិនធ្លាប់រួមភេទ: ០		
១៧	រយៈពេល ៣ខែកន្លងមកនេះ តើនាងធ្លាប់បានរួមភេទ ជាមួយមិត្តប្រុស/សង្សាររបស់នាងប៉ុន្មានដងដែរ?	ចំនួន ……………. ដង<br>មិនធ្លាប់រួមភេទ: ០		
១៨	ក្នុងរយៈពេល ៣ខែកន្លងមក នេះ តើនាងបានប្រើស្រោមអនាម័យយ៉ាងណាដែរ នៅពេលរួមភេទជាមួយ មិត្តប្រុស/សង្សាររបស់នាង **(សង្សារចុងក្រោយបង្អស់ ឬ សង្សារបច្ចុប្បន្ន)**	មិនធ្លាប់រួមភេទ: ០<br>គ្រប់ពេល (១០០%): ១<br>ញឹកញាប់ (៦០-៩៩ %): ២<br>ម្តងម្កាល (១-៥៩%): ៣<br>មិនដែលប្រើសោះ(០%): ៤		
១៩	ក្នុងរយៈពេល ៣ខែកន្លងមក នេះ តើនាងបានប្រើស្រោមអនាម័យយ៉ាងណាដែរ ជាមួយប្តីរបស់នាង?	មិនធ្លាប់រួមភេទ: ០<br>គ្រប់ពេល (១០០%): ១<br>ញឹកញាប់ (៦០-៩៩ %): ២<br>ម្តងម្កាល (១-៥៩%): ៣		

		មិនដែលប្រើសោះ(0%): ៤		
២០	ក្នុងរយៈពេល ១២ខែកន្លងមក តើនាងធ្លាប់ទទួលអំណោយ ឬ ប្រាក់ពីប្រុសៗដើម្បីប្តូរការរួមភេទដែរឬទេ? **(មិនគិតរួមជាមួយមិត្តប្រុស/សង្សារ/ប្តី)**	មិនដែលរួមភេទ: 0<br>១. ធ្លាប់: ១<br>មិនធ្លាប់: ២	ទៅសំនួរ<br><br>ទៅសំនួរ	៣៤<br><br>៣៤
២១	តើក្នុង ១សប្តាហ៍ នាងរួមភេទជាមួយភ្ញៀវប៉ុន្មាននាក់គិតជាមធ្យម? **(មិនគិតរួមជាមិត្តប្រុស/សង្សារ/ប្តី)**	ចំនួនភ្ញៀវ...............នាក់<br>មិនបានរួមភេទ: 0		
២២	នៅថ្ងៃធ្វើការចុងក្រោយរបស់នាង តើនាងរួមភេទជាមួយភ្ញៀវបាន ចំនួនប៉ុន្មាននាក់? **(កំឡុងពេល១២ខែចុងក្រោយ)**	ចំនួនភ្ញៀវ.......................នាក់<br>មិនបានរួមភេទ/អត់ភ្ញៀវ: 0		
២៣	នៅពេលរួមភេទជាមួយភ្ញៀវចុងក្រោយបំផុត តើនាងបានប្រើស្រោមអនាម័យដែរឬទេ? **(កំឡុងពេល១២ខែចុងក្រោយ)**	ចាស៎: ១<br>មិនបានប្រើ: ២		
២៤.	ក្នុងរយៈពេល ១សប្តាហ៍កន្លងមក តើនាងបានប្រើស្រោមអនាម័យយ៉ាងណាដែរជាមួយភ្ញៀវរបស់នាង?	មិនបានរួមភេទជាមួយភ្ញៀវ: 0<br>គ្រប់ពេល(១00%): ១<br>ញឹកញាប់ (៦0-៩៩%): ២<br>ម្តងម្កាល(១-៥៩%): ៣<br>មិនដែលប្រើសោះ(0%): ៤		
២៥	ក្នុងរយៈពេល ៣ខែកន្លងមក តើនាងបានប្រើស្រោមអនាម័យយ៉ាងណាដែរ នៅពេលរួមភេទ ជាមួយភ្ញៀវរបស់នាង?	មិនបានរួមភេទជាមួយភ្ញៀវ: 0<br>គ្រប់ពេល(១00%): ១<br>ញឹកញាប់ (៦0-៩៩%): ២<br>ម្តងម្កាល(១-៥៩%): ៣<br>មិនដែលប្រើសោះ(0%): ៤		

២៦	តើបច្ចុប្បន្ននេះនាងមានភ្ញៀវរួមភេទទៀងទាត់(ម៉ូយ)ដែរឬទេ? **(គេងជាមួយនាងលើសពី ៥ដង)**	ចាស់: ១<br>មិនមាន: ២	ទៅសំនួរ	២៨
២៧	ក្នុង ១ខែកន្លងមក តើនាងបានប្រើស្រោមអនាម័យជាមួយភ្ញៀវទៀងទាត់(ម៉ូយ) យ៉ាងណាដែរ?	មិនបានរួមភេទជាមួយភ្ញៀវ: ០<br>គ្រប់ពេល(១០០%): ១<br>ញឹកញាប់ (៦០-៩៩%): ២<br>ម្តងម្កាល(១-៥៩%): ៣<br>មិនដែលប្រើសោះ(០%): ៤		
២៨	ក្នុងរយៈពេល ១២ខែកន្លងមក តើនាងបានដែលរួមភេទជាមួយអ្នកចិញ្ចឹមដែរឬទេ?	មិនមានអ្នកចិញ្ចឹម: ០<br>ចាស់: ១<br>ទេ: ២	ទៅសំនួរ<br><br>ទៅសំនួរ	៣០<br><br>៣០
២៩	ក្នុងរយៈពេល ៣ខែកន្លងមក តើនាងបានប្រើស្រោមអនាម័យជាមួយអ្នកចិញ្ចឹមញឹកញាប់យ៉ាងណាដែរ? **(អ្នកចិញ្ចឹមចុងក្រោយ ឬ បច្ចុប្បន្ន)**	មិនបានរួមភេទទេ: ០<br>គ្រប់ពេល(១០០%): ១<br>ញឹកញាប់ (៦០-៩៩%): ២<br>ម្តងម្កាល(១-៥៩%): ៣<br>មិនដែលប្រើសោះ(០%): ៤		
៣០	ក្នុងរយៈពេល ៣ខែកន្លងមក តើនាងធ្លាប់រួមភេទតាមរន្ធគូទដែរឬទេ?	មិនដែលរួមភេទ: ០<br>ចាស់ធ្លាប់: ១<br>មិនធ្លាប់: ២		
៣១	ក្នុងរយៈពេល ៣ខែកន្លងមក តើនាងធ្លាប់រួមភេទដោយប្រើមាត់ដែរឬទេ?	មិនដែលរួមភេទ: ០<br>ចាស់ធ្លាប់: ១<br>មិនធ្លាប់: ២		
៣២	ក្នុងរយៈពេល ៣ខែកន្លងមក តើនាងធ្លាប់រួមភេទដោយមិនបានប្រើស្រោមអនាម័យដែរឬទេ ក្រៅពីប្តី?	មិនដែលរួមភេទ/មិនធ្លាប់រួមភេទជាមួយភ្ញៀវ: ០<br>ចាស់ធ្លាប់: ១<br>មិនធ្លាប់: ២	ទៅសំនួរ<br><br>ទៅសំនួរ	៣៤<br><br>៣៤
៣៣	ហេតុអ្វីបានជាអ្នកមិនប្រើស្រោមអនាម័យ នៅពេលរួម	ចង់មានផ្ទៃពោះ: ១<br>គិតថា គាត់អត់មានជម្ងឺកាមរោគ ឬមេរោគអេដស៍: ២		

	ភេទ? (**សូមអានចម្លើយទាំងអស់ ចម្លើយអាចមានច្រើន**)	គាត់បញ្ចុះបញ្ចូល មិនអោយប្រើស្រោម: ៣<br>គាត់បង្ខំ មិនអោយប្រើស្រោម: ៤<br>គាត់អោយលុយច្រើនបើអត់ពាក់ស្រោម: ៥<br>ស្មោះស្ម័គ្រ/យល់ចិត្តគ្នា: ៦<br>អត់មានស្រោមអនាម័យ: ៧<br>វង្វេងដោយសារប្រើថ្នាំញៀន ឬ គ្រឿងស្រវឹង: ៨<br>ផ្សេងៗ (សូមបញ្ជាក់).........................: ៩		
៣៤	ក្នុងកំឡុងពេលនាងមករដូវនៅខែចុងក្រោយ តើនាងឈប់រួមភេទប៉ុន្មានថ្ងៃ?	ចំនួន.......... ថ្ងៃ<br>មិនបានឈប់: ០<br>មិនដែលរួមភេទ: ៩៦<br>មិនមានរដូវ: ៩៧		
៣៥	ក្នុងរយៈពេល ៣ខែកន្លងមក តើនាងមានបានទទួលការអប់រំ ស្តីពីជំងឺអេដស៍/មេរោគអេដស៍ ឬជំងឺកាមរោគតាមមធ្យោបាយណាខ្លះ? (**សូមអានចំលើយ, ចំលើយច្រើន**)	មិនដែលបានទទួលព័ត៌មាន ការអប់រំ: ០<br>ទូរទស្សន៍: ១<br>វិទ្យុ: ២<br>កាសែត (សារព័ត៌មាន): ៣<br>ផ្ទាំងរូបភាពធំៗ (បៀលបត): ៤<br>ផ្ទាំងរូបភាពសំរាប់អប់រំ: ៥<br>កូនសៀវភៅអប់រំ: ៦<br>ការពិភាក្សាជាក្រុមជាមួយបុគ្គលិកចុះផ្ទាល់: ៧<br>ការផ្តល់ប្រឹក្សាជាបុគ្គល: ៨<br>អ្នកអប់រំបន្ត: ៩<br>ផ្សេងៗ(សូមបញ្ជាក់) ....: ១០		
៣៦	ក្នុងរយៈពេល ៣ខែកន្លងមក តើនាងមានបានទទួលព័ត៌មានការអប់រំ ស្តីពីជំងឺអេដស៍/ជំងឺកាមរោគ តាមរយៈបុគ្គលិកអប់រំណាខ្លះ?<br>(**សូមអានចំលើយ, ចំលើយច្រើន**)	មិនដែលបានទទួលព័ត៌មាន ការអប់រំ: ០<br>បុគ្គលិក គ្លីនិកកាមរោគ ១<br>បុគ្គលិក គ្លីនិកអង្គការ ២<br>បុគ្គលិកចុះអប់រំផ្ទាល់ ៣<br>អ្នកអប់រំបន្ត/មិត្តអប់រំមិត្ត ៤<br>បុគ្គលិកមជ្ឈមណ្ឌលធ្វើតេស្តឈាមដោយស្ម័គ្រចិត្ត និងរក្សាការសំងាត់ ៥<br>គ្រូពេទ្យឬបុគ្គលិកនៅគ្លីនិកឯកជន ៦		

		ផ្សេងៗ (សូមបញ្ជាក់) ៧		
៣៧	ក្នុងរយៈពេល ១២ខែកន្លងមក តើគេដែលអោយស្រោមអនាម័យនាងដែរឬទេ?	ចាស់ ១<br>ទេ ២		
៣៨	ក្នុងរយៈពេល ៣ខែកន្លងមក តើនាងដែលបានទទួលស្រោមអនាម័យដោយមិនបាច់ចំណាយប្រាក់យ៉ាងណាដែរ?	មិនបានទទួលស្រោមអនាម័យ; ០<br>បានទទួលស្រោមអនាម័យដោយមិនបាច់ចំណាយប្រាក់ ១<br>ពេលខ្លះទទួលបានមិនបាច់ចំណាយប្រាក់ពេលខ្លះទិញ ២<br>តែងតែទិញស្រោមអនាម័យ ៣<br>ស្រោមអនាម័យរបស់ភ្ញៀវ ឬមិត្តប្រុស ៤<br>មិនទទួលស្រោមអនាម័យដែលមិនបាច់ចំណាយប្រាក់ ៥		
៣៩	ក្នុងរយៈពេល ៣ខែកន្លងមក តើនាងបានទទួលស្រោមអនាម័យមកពីកន្លែងណាខ្លះ?<br>(អាចមានចំលើយច្រើន)	មិនដែលប្រើស្រោមអនាម័យៈ ០<br>នៅកន្លែងដែលខ្ញុំធ្វើការៈ ១<br>បានពីភ្ញៀវ ឬមិត្តប្រុសៈ ២<br>ពីបុគ្គលិកចុះអប់រំផ្ទាល់ៈ ៣<br>អ្នកអប់រំបន្ត/មិត្តអប់រំមិត្តៈ ៤<br>ពីកន្លែងលក់ថ្នាំ ៈ៥<br>គ្លីនិកសុខភាពគ្រួសារ/មណ្ឌលសុខភាព/មន្ទីរពេទ្យរដ្ឋៈ ៦<br>គ្លីនិករបស់អង្គការ ៧<br>ផ្សេងៗ (សូមបញ្ជាក់)............៨		

ផ្នែកទី៣: ការប្រព្រឹត្តហឹង្សាផ្លូវភេទ

លរ	សំណួរ	កូដ ចម្លើយ	រំលង	កូដ
៤០a	តើប្ដី/ដៃគូរួមភេទរបស់នាងធ្លាប់ប្រើអំពើហឹង្សាវាយធ្វើបាបបង្ខិតបង្ខំលើរាងកាយនាង ដែរឬទេ?	ចាស់; ១<br>ទេ; ២<br>មិនឆ្លើយ; ៩៨		

៤០b	តើអំពើហឹង្សាទាំងនេះ វាកើតមានញឹកញាប់យ៉ាង ណាក្នុងរយៈពេល ១២ខែកន្លងមក?	ញឹកញាប់; ១<br>ម្តងម្កាល; ២<br>មិនដែលសោះ; ៣<br>មិនឆ្លើយ; ៩៨		
៤១a	ក្រៅពីប្តី/ដៃគូរួមភេទរបស់នាង តើមានអ្នកណាផ្សេងធ្លាប់ដែលប្រើអំពើហឹង្សាវាយធ្វើបាបបង្ខិតបង្ខំលើរាងកាយនាងដែរឬទេ?	ចាស៎; ១<br>ទេ; ២<br>មិនឆ្លើយ; ៩៨		
៤១b	តើអំពើហឹង្សាទាំងនេះ វាកើតមានញឹកញាប់យ៉ាង ណាក្នុងរយៈពេល ១២ខែកន្លងមក?	ញឹកញាប់; ១<br>ម្តងម្កាល; ២<br>មិនដែលសោះ; ៣<br>មិនឆ្លើយ; ៤		
៤២a	តើនាងធ្លាប់ត្រូវបានប្តី/ដៃគូរួមភេទរបស់នាង ប្រើអំពើហឹង្សាវាយធ្វើបាបបង្ខិតបង្ខំលើរាងកាយ នាង នៅពេលគាត់ចង់រួមភេទ ហើយនាងមិនចង់រួមភេទដែរឬទេ?	ចាស៎; ១<br>ទេ; ២<br>មិនឆ្លើយ; ៩៨		
៤២b	តើអំពើហឹង្សាទាំងនេះ វាកើតមានញឹកញាប់យ៉ាង ណាក្នុងរយៈពេល ១២ខែកន្លងមក?	ញឹកញាប់; ១<br>ម្តងម្កាល; ២<br>មិនដែលសោះ; ៣<br>មិនឆ្លើយ; ៤		
៤៣a	ក្រៅពីប្តី/ដៃគូរួមភេទរបស់នាង តើនាងធ្លាប់ត្រូវបានអ្នកណាផ្សេងប្រើអំពើហឹង្សាវាយធ្វើបាបបង្ខិតបង្ខំលើរាងកាយនាង នៅពេលគាត់ចង់រួមភេទ	ចាស៎; ១<br>ទេ; ២<br>មិនឆ្លើយ; ៩៨		

	ហើយនាងមិនចង់រួមភេទដែរឬទេ?			
៤៣b	តើអំពើហឹង្សាទាំងនេះវាកើតមានញឹកញាប់យ៉ាងណាក្នុងរយៈពេល ១២ខែកន្លងមក?	ញឹកញាប់; ១<br>ម្តងម្កាល; ២<br>មិនដែលសោះ; ៣<br>មិនឆ្លើយ; ៤		
៤៤a	តើនាងធ្លាប់បានត្រូវគេបង្ខំរួមភេទជាមួយបុរសជាក្រុម*នៅពេលដែលនាងមិនចង់រួមភេទដែរឬទេ?<br>*បុរសចាប់ពីពីរនាក់ឡើងទៅ	ចាស៎; ១<br>ទេ; ២<br>មិនឆ្លើយ; ៩៨		
៤៤b	តើអំពើហឹង្សាទាំងនេះវាកើតមានញឹកញាប់យ៉ាងណាក្នុងរយៈពេល ១២ខែកន្លងមក?	ញឹកញាប់; ១<br>ម្តងម្កាល; ២<br>មិនដែលសោះ; ៣<br>មិនឆ្លើយ; ៤		
ប្រសិនបើចម្លើយ "ចាស៎" ក្នុងសំណួរ ៤០-៤៤ សូមបន្តៈ				
៤៥	តើនាងបានទៅស្វែងរកគ្រូពេទ្យជួយ ឬព្យាបាលរបួសដែរឬទេ? ក្រោយពីមានអំពើហឹង្សាបង្ខំរួមភេទពីនរណាម្នាក់?	ចាស៎; ១<br>ទេ; ២<br>មិនឆ្លើយ; ៩៨		

<u>ផ្នែកទី៤: ការប្រើប្រាស់គ្រឿងញៀន</u>

**លរ**	**សំណួរ**	**កូដ ចម្លើយ**	**រំលង**	**កូដ**
៤៦	មានអ្នកខ្លះបានប្រើប្រាស់គ្រឿងញៀន ដើម្បីមូលហេតុផ្សេងៗ ដូចជា ការសប្បាយ ដើម្បីបង្កើនកំលាំង តើនាងធ្លាប់បានប្រើប្រាស់គ្រឿងញៀនដែរ	ចាស៎ធ្លាប់; ១<br>មិនធ្លាប់; ២<br>គ្មានចម្លើយ: ៩៨<br>មិនដឹង: ៩៩	ទៅសំនួរ	៤៩

	ឬទេ? **(សូមរំលឹកអ្នកចូលរួមថា ចម្លើយរបស់គាត់គឺឯកជននឹងសម្ងាត់)**			
៤៧	ក្នុងរយៈ ១២ខែចុងក្រោយនេះ តើថ្នាំប្រភេទណាខ្លះដែលនាងបានប្រើ?<br>**(សូមអានចំលើយ អាចមានចំលើយច្រើន)**	មិនដែលប្រើគ្រឿងញៀនក្នុងមួយឆ្នាំកន្លងទៅនេះ: 0<br>ហេរូអ៊ីន: ១<br>ម្សៅអំហ្វេតាមីន (ទឹកកក យ៉ាម៉ា): ២<br>អ៊ិចតាស៊ី: ៣<br>ថ្នាំសំរាប់គេងលក់ (Valium, Diazepam): ៤<br>ហ៊ិតការ: ៥<br>កញ្ឆា: ៦<br>គ្រឿងញៀនផ្សេងទៀត (សូមបញ្ជាក់)..............................: ៧<br>គ្មានចម្លើយ: ៩៨		
៤៨	ក្នុងរយៈពេល ១២ខែកន្លងមក តើនាងធ្លាប់បានចាក់ថ្នាំញៀនដែរឬទេ?	ចាស៎ ធ្លាប់: ១<br>ទេ មិនធ្លាប់: ២<br>គ្មានចម្លើយ: ៩៨<br>មិនដឹង: ៩៩		
៤៩	ក្នុងរយៈពេល ១សប្តាហ៍កន្លងមក តើនាងផឹកគ្រឿងស្រវឹង ញឹកញាប់ប៉ុណ្ណាដែរពេលកំពុងបំរើការងាររបស់នាង?	ចំនួនថ្ងៃ ....ថ្ងៃ<br>មិនដែលសោះ: 0<br>រៀងរាល់ថ្ងៃ: ៧		
៥០	ក្នុងរយៈពេល ១សប្តាហ៍កន្លងមក ជាធម្មតា តើនាងផឹកគ្រឿងស្រវឹងច្រើនយ៉ាងណាដែរពេលកំពុងធ្វើការងារ? (គ្រឿងស្រវឹងមួយមានន័យថា ស្រាបៀរមួយកំប៉ុង = មួយដប = ស្រាមួយកែវ)	ចំនួនគ្រឿងស្រវឹង ....<br>មិនដែលផឹក: 0<br>គ្រឿងស្រវឹង៩៦កំប៉ុង/កែវ ឬ ច្រើនជាង: ៩៦<br>គ្រឿងស្រវឹងតិចជាង ១កំប៉ុង/កែវ: ៩៧		

ផ្នែកទី៤: ឥរិយាបថក្នុងការប្រើប្រាស់សេវាសុខាភិបាល

**លរ**	**សំណួរ**	**កូដ ចម្លើយ**	**រំលង**	**កូដ**
៥១	ក្នុងរយៈពេល ១២ខែកន្លងមក តើនាងមានធ្លាប់មានដំបៅនៅប្រដាប់ភេទដែរឬទេ?	ធ្លាប់: ១<br>មិនធ្លាប់: ២		
៥២	ក្នុងរយៈពេល ១២ខែកន្លងមក តើនាងមានធ្លាប់កើតជំងឺសេមាន់ដែររឺទេ?	ធ្លាប់: ១<br>មិនធ្លាប់: ២		
៥៣	ក្នុងរយៈពេល ១២ខែកន្លងមក តើនាងមានធ្លាប់ធ្លាក់សតាមទ្វារមាសខុសធម្មតាដែររឺទេ?	ធ្លាប់: ១<br>មិនធ្លាប់: ២		
៥៤	ក្នុងរយៈពេល ១២ខែកន្លងមក តើនាងមានធ្លាប់ឈឺចាប់ផ្នែកខាងក្រោមនៃពោះដែរឬទេ? (សូមចង្អុលចំផ្នែកខាងក្រោមនៃពោះ)	ធ្លាប់: ១<br>មិនធ្លាប់: ២		
៥៥	ក្នុងរយៈពេល ១២ខែកន្លងមក តើនាងគិតថាខ្លួនមានឆ្លងជំងឺកាមរោគដែរឬទេ?	ធ្លាប់: ១<br>មិនធ្លាប់: ២		
៥៦	ក្នុងរយៈពេល ១២ខែកន្លងមក ពេលនាងមានជំងឺកាមរោគចុងក្រោយនេះ តើនាងទៅព្យាបាលនៅកន្លែងណា? (ចំលើយមានតែ១)	មិនដែលមានជំងឺកាមរោគ: ០<br>មណ្ឌលសុខភាព/ពេទ្យរដ្ឋ: ១<br>គ្លីនិកសុខភាពគ្រួសារ (គ្លីនិកកាមរោគរដ្ឋ): ២<br>គ្លីនិកកាមរោគរបស់អង្គការ: ៣<br>គ្លីនិកឯកជន: ៤<br>គ្រូខ្មែរ/គ្រូបុរាណ: ៥<br>ទិញថ្នាំដែលគេផ្សំអោយនៅឱសថស្ថាន: ៦<br>មិនព្យាបាលសោះ: ៧<br>ផ្សេងៗ(សូមបញ្ជាក់)............................: ៨	ទៅសំនួរ	៥៨

៥៧	ក្នុងរយៈពេល ១២ខែកន្លងមក តើនាងបានឈប់រួមភេទ ចំនួនប៉ុន្មានថ្ងៃ? ពេលដែលអ្នកមាន ជំងឺកាមរោគចុងក្រោយ (សូមអានចំលើយ, ចំលើយមានតែ១)	មិនដែលរួមភេទ: ០<br>មិនបានឈប់រួមភេទ: ១<br>១ថ្ងៃ ទៅ ៣ថ្ងៃ: ២<br>៤ថ្ងៃ ទៅ ១០ថ្ងៃ: ៣<br>រហូតដល់ព្យាបាលចប់: ៤<br>រហូតដល់ជាសះស្បើយ: ៥		
៥៨	ពេលបច្ចុប្បន្ន តើនាងប្រើមធ្យោបាយ ឬ វិធីសាស្ត្រអ្វីដើម្បីបង្ការកុំអោយមានកូន? (អាចមានចំលើយច្រើន)	មិនដែលរួមភេទ: ០<br>មិនប្រើវិធីសាស្ត្រពន្យាកំណើតណាមួយឡើយ: ១<br>ចងដៃស្បូន: ២<br>ចងបំពង់ទឹកកាមបុរស: ៣<br>លេបថ្នាំរាល់ថ្ងៃ: ៤<br>លេបថ្នាំរាល់ខែ: ៥<br>ចាក់ថ្នាំ: ៦<br>ដាក់កងក្នុងស្បូន: ៧<br>ដាក់កងដៃ: ៨<br>ស្រោមអនាម័យបុរស: ៩<br>ស្រោមអនាម័យស្ត្រី: ១០<br>បំបៅដោះកូន: ១១<br>ថ្នាំរងាប់មេជីវិតបុរស: ១២<br>វិធីប្រតិទិន: ១៣<br>ដកលិង្គចេញក្រៅមុនពេលចេញទឹកកាម (ចាក់ទឹកក្រៅពាង): ១៤<br>ថ្នាំពន្យាកំណើតក្រោយការរួមភេទ: ១៥<br>ផ្សេងៗ(សូមបញ្ជាក់) ....: ១៦		

ផ្នែកទី៦: ចំណេះដឹងពីស្ថានភាពជំងឺអេដស៍ និងជំងឺកាមរោគ

លរ	សំណួរ	កូដ ចម្លើយ	រំលង	កូដ
៥៩	តើនាងដឹងថាត្រូវទៅទីណាទេ ប្រសិនបើនាងត្រូវការធ្វើតេស្តឈាមរកមេរោគអេដស៍?	ចាស់ដឹង: ១<br>មិនដឹង: ២		

៦០	ក្នុងរយៈពេល ១២ខែកន្លងមក តើនាងធ្លាប់ធ្វើតេស្តឈាមរកមេរោគអេដស៍ដែរឬទេ?	ធ្លាប់: ១<br>មិនធ្លាប់: ២		
៦១	ប្រសិនបើនាងធ្លាប់ធ្វើតេស្តឈាមរកមេរោគអេដស៍ តើរយៈពេលប៉ុន្មានខែកន្លងមកហើយ?	ចំនួន................ ខែ<br>មិនដែលធ្វើតេស្តឈាម: ០<br>តិចជាង ឬស្មើ១ខែ: ១<br>លើសពី១ឆ្នាំមុន: ១៣		
៦២	តើនាងធ្វើតេស្តឈាមរកមេរោគអេដស៍ចុងក្រោយបង្អស់នៅកន្លែងណា?	មិនដែលធ្វើតេស្តឈាម: ០<br>គ្លីនិកឯកជន ឬមន្ទីរពិសោធន៍: ១<br>មន្ទីរពេទ្យរដ្ឋ: ២<br>មជ្ឈមណ្ឌលធ្វើតេស្តឈាមដោយស្ម័គ្រចិត្ត និងរក្សាការសំងាត់ (រដ្ឋ): ៣<br>មជ្ឈមណ្ឌលធ្វើតេស្តឈាមដោយស្ម័គ្រចិត្ត និងរក្សាការសំងាត់(អង្គការ): ៤<br>ការធ្វើតេស្តឈាមចល័ត: ៥<br>ឱសថស្ថាន: ៦<br>ផ្សេងៗ(សូមបញ្ជាក់)..........................: ៧	ទៅសំនួរ	៦៩
៦៣	តើនាងបានទទួលលទ្ធផលនៃការធ្វើតេស្តឈាមរកមេរោគអេដស៍ ចុងក្រោយដែររឺទេ? (នាងមិនចាំបាច់ប្រាប់ខ្ញុំពីលទ្ធផលទេ)	បាន: ១<br>មិនបាន: ២		
៦៤	តើអ្នកមានអារម្មណ៍មិនទើសទាល់ ក្នុងការប្រាប់ខ្ញុំពីលទ្ធផលនៃការធ្វើតេស្តឈាមរកមេរោគអេដស៍ចុងក្រោយ របស់អ្នកដែរឬទេ?	ចាស៎: ១<br>ទេ: ២	ទៅសំនួរ	៦៩
៦៥	ប្រសិនបើនាងមានអារម្មណ៍មិនទើសទាល់ក្នុងការប្រាប់ខ្ញុំ តើលទ្ធផលតេស្តឈាមរបស់នាងយ៉ាងដូចម្តេចដែរ? (រំលឹកដល់	លទ្ធផលតេស្តរកមេរោគអេដស៍វិជ្ជមាន: ១<br>លទ្ធផលតេស្តរកមេរោគអេដស៍មិនច្បាស់: ២<br>លទ្ធផលតេស្តរកមេរោគអេដស៍អវិជ្ជមាន: ៣	ទៅសំនួរ	៦៩

	អ្នកឆ្លើយថា ចំលើយរបស់នាងនឹងត្រូវទុកជាការសំងាត់)			
៦៦	បើលទ្ធផលតេស្តឈាមរកមេរោគអេដស៍របស់អ្នកវិជ្ជមាន តើនាងបានធ្វើតេស្តឈាម ប៉ុន្មានដងដើម្បីបញ្ជាក់ពីលទ្ធផលនេះ?	ចំនួន .... ដង		
៦៧	បើលទ្ធផលតេស្តឈាមរកមេរោគអេដស៍របស់អ្នកវិជ្ជមាន តើនាងបានទៅចុះឈ្មោះនៅមណ្ឌលសេវាថែទាំ និងព្យាបាលជំងឺអេដស៍ដែរឬទេ?	ចាស៎: ១<br>ទេ: ២		
៦៨	បើលទ្ធផលតេស្តរកមេរោគអេដស៍របស់អ្នកវិជ្ជមាន តើនាងកំពុងបានទទួលឱសថប្រឆាំងនឹងមេរោគអេដស៍ដែរឬទេ?	ចាស៎: ១<br>ទេ: ២		
៦៩	តើសេវាព្យាបាលដោយប្រើថ្នាំពន្យាជីវិតអាចរកបាននៅក្នុងប្រទេសកម្ពុជា សំរាប់អ្នកផ្ទុកមេរោគអេដស៍ ដែរឬទេ?	ចាស៎: ១<br>ទេ: ២<br>មិនដឹង: ៣		
៧០	ក្នុងរយៈពេល ១២ខែកន្លងមក តើអ្នកឧស្សាហ៍ទៅគ្លីនិកពិនិត្យមើលសុខភាពផ្លូវភេទបានប៉ុន្មានដង?	.... ដង<br>មិនដែលបានទៅសោះ៖ ០	ទៅសំនួរ	៧៣
៧១	ក្នុងរយៈពេល ១ឆ្នាំកន្លងមក តើនាងបានទៅគ្លីនិក ដើម្បីឱ្យគេពិនិត្យសុខភាពផ្លូវភេទលើកចុងក្រោយ បានប៉ុន្មានខែហើយ?	.... ខែ<br>មិនដែលព្យាបាល: ០		
៧២	តើគ្លីនិកមួយណាដែលនាងបានទៅលើកចុងក្រោយ? (សូមអានចំលើយ មានចំលើយតែ១)	គ្លីនិកសុខភាពគ្រួសារ(គ្រប់គ្រងដោយ NCHADS): ១<br>គ្លីនិករបស់ក្រសួងសុខាភិបាល/រដ្ឋ: ២		

		គ្លីនិកអង្គការគ្រប់គ្រងដោយ RHAC, MSIC, MEC,PSF: ៣<br>គ្លីនិកអង្គការគ្រប់គ្រងដោយអង្គការផ្សេងទៀត: ៤<br>គ្លីនិកឯកជន: ៥<br>មិនស្គាល់ឈ្មោះគ្លីនិក: ៦<br>គ្លីនិកផ្សេងទៀត (សូមបញ្ជាក់)................ : ៧		
៧៣	នៅពេលដែលនាងរួមភេទជាមួយដៃគូស្មោះត្រង់តែម្នាក់ដែលគ្មានមេរោគអេដស៍ តើវាអាចធ្វើឱ្យនាងឆ្លងមេរោគអេដស៍ដែរឬទេ?	មិនដឹង: 0<br>ចាស វាអាច: ១<br>ទេ មិនអាច: ២		
៧៤	តើស្រោមអនាម័យអាចការពារការឆ្លងមេរោគអេដស៍ដែរឬទេ?	មិនដឹង: 0<br>ចាស វាអាច: ១<br>ទេ មិនអាច: ២		
៧៥	តើអ្នកដែលយើងមើលទៅឃើញមានសុខភាពល្អ អាចចម្លងមេរោគអេដស៍ដែរឬទេ?	មិនដឹង: 0<br>ចាស វាអាច: ១<br>ទេ មិនអាច: ២		
៧៦	តើសត្វមូសអាចចម្លងមេរោគអេដស៍ពីមនុស្សដែលមានផ្ទុកមេរោគអេដស៍ ទៅកាន់មនុស្សដែល គ្មានផ្ទុកមេរោគអេដស៍ដែរឬទេ?	មិនដឹង: 0<br>ចាស វាអាច: ១<br>ទេ មិនអាច: ២		
៧៧	តើការញ៉ាំជាមួយអ្នកដែលមានផ្ទុកមេរោគអេដស៍ អាចចម្លងមេរោគអេដស៍ដែរឬទេ?	មិនដឹង: 0<br>ចាស វាអាច: ១<br>ទេ មិនអាច: ២		

ចប់

<u>សូមថ្លែងអំណរគុណចំពោះការចូលរួមឆ្លើយ</u>
